# Comprehensive Analysis of Differentially Expressed lncRNA, circRNA and mRNA and Their ceRNA Networks in Mice With Severe Acute Pancreatitis

**DOI:** 10.3389/fgene.2021.625846

**Published:** 2021-01-28

**Authors:** Bing Wang, Jun Wu, Qilin Huang, Xiaohui Yuan, Yi Yang, Wen Jiang, Yi Wen, Lijun Tang, Hongyu Sun

**Affiliations:** ^1^Department of General Surgery & Pancreatic Injury and Repair Key Laboratory of Sichuan Province, The General Hospital of Western Theater Command, Chengdu, China; ^2^College of Medicine, Southwest Jiaotong University, Chengdu, China; ^3^Laboratory of Basic Medicine, The General Hospital of Western Theater Command, Chengdu, China

**Keywords:** competitive endogenous RNA, long noncoding RNA, circular RNA, mRNA, severe acute pancreatitis

## Abstract

Severe acute pancreatitis (SAP) is an acute digestive system disease with high morbidity mortality and hospitalization rate worldwide, due to various causes and unknown pathogenesis. In recent years, a large number of studies have confirmed that non-coding RNAs (ncRNAs) play an important role in many cellular processes and disease occurrence. However, the underlying mechanisms based on the function of ncRNAs, including long noncoding RNA (lncRNA) and circular RNA (circRNA), in SAP remain unclear. In this study, we performed high-throughput sequencing on the pancreatic tissues of three normal mice and three SAP mice for the first time to describe and analyze the expression profiles of ncRNAs, including lncRNA and circRNA. Our results identified that 49 lncRNAs, 56 circRNAs and 1,194 mRNAs were differentially expressed in the SAP group, compared with the control group. Furthermore, we performed Gene Ontology (GO) and Kyoto Encyclopedia of Genes and Genomes (KEGG) analysis of differentially expressed lncRNAs and circRNAs, and found that the functions of the parental genes are enriched in the calcium-regulated signaling pathway, NF-κB signaling pathway, autophagy and protein digestion and absorption processes, which are closely related to the central events in pathogenesis of SAP. We also constructed lncRNA/circRNA-miRNA-mRNA networks to further explore their underlying mechanism and possible relationships in SAP. We found that in the competitive endogenous RNA (ceRNA) networks, differentially expressed lncRNAs and circRNAs are mainly involved in the apoptosis pathway and calcium signal transduction pathway. In conclusion, we found that lncRNAs and circRNAs play an important role in the pathogenesis of SAP, which may provide new insights in further exploring the pathogenesis of SAP and seek new targets for SAP.

## Introduction

Acute pancreatitis (AP) is a self-digestive disease caused by the activation of trypsin in the pancreas due to various causes. It is one of the main reasons for hospitalization due to gastrointestinal diseases. 20% of AP can further develop into severe acute pancreatitis (SAP), and due to local or systemic complications and invasive intervention, the mortality rate is up to 30% (Trikudanathan et al., 2019). A large number of studies have found that pathological calcium signaling, premature activation of trypsinogen in acinar cells, endoplasmic reticulum stress, impaired autophagy and mitochondrial dysfunction play important roles in the pathogenesis of SAP (Lee and Papachristou, 2019). However, its pathogenesis is still unclear. At present, it is recognized that the treatment of SAP worldwide is to adopt a staged, multidisciplinary, and ascending symptomatic treatment strategy, lacking of more effective treatment methods now (da Costa et al., 2014). Therefore, it is necessary to further explore the unknown molecular mechanism and discover potential biomarkers and new therapeutic targets.

More and more studies have shown that non-coding RNAs (ncRNAs), including long noncoding RNA (lncRNA), circular RNA (circRNA) and microRNA (miRNA), exert great influence on the occurrence and development of various diseases, including heart diseases (De Majo and De Windt, 2018), liver diseases (Ma et al., 2019), kidney diseases (Cao et al., 2018), lung diseases (Wang et al., 2019) and so on, and the expression of ncRNAs can be regulated strictly under physiological conditions (Beermann et al., 2016). LncRNA refers to an RNA molecule with a length of more than 300 nucleotides (nt) without an open reading frame that encodes a protein. It is reported that lncRNA in cells accounts for the abundance of about 70–98%, which is a regulator of almost every cellular process (Marchese et al., 2017). Studies have shown that some lncRNAs are involved in the pathophysiological process of SAP, including the regulation of lipid metabolism (Tontonoz et al., 2017), the maintenance of intact intestinal barrier (Zou et al., 2016), and the autophagy flux (Antonucci et al., 2015). However, there is no comprehensive analysis on the role of lncRNA in SAP.

CircRNA is an endogenous non-coding RNA with a covalent closed loop structure, usually from RNA reverse splicing and exon or intron circularization. CircRNA is highly abundant in eukaryotes and highly conservative in evolution. The expression of circRNA is tissue-specific and cell-specific, and circRNA participates in many diseases (Memczak et al., 2013), such as pancreatic ductal adenocarcinoma (Xiao, 2020), diabetes (Bai et al., 2019), cardiovascular diseases (Altesha et al., 2019) and so on. In our previous study, we found that compared with normal pancreas mice, miR-21-3p was significantly higher in mice with SAP, which was negatively correlated with circZFP644. It is speculated that circZFP644 may be used as a sponge for miR-21-3p to regulate its target genes, ultimately leading to necrotizing pancreatitis (Yang et al., 2020).

Both lncRNA and circRNA can function through sponging with miRNA to sequester miRNAs, thereby preventing the interaction of miRNAs with target mRNA and exerting competitive endogenous RNA (ceRNA) activity (Salmena et al., 2011; Tang et al., 2017). The ceRNA activity forms a large-scale and complex post-transcriptional regulatory network in the entire transcriptome, which greatly expands the functional genetic information in the human genome. In the ceRNA mechanism, lncRNA, circRNA, and mRNA have effective crosstalk with miRNA, and they are regulated in various pathophysiological processes of plants and animals.

Little is known about the function of lncRNA and circRNA in SAP, so it is necessary to analyze the lncRNA and circRNA comprehensively, and explore the role of lncRNA/circRNA-miRNA-mRNA ceRNA network in SAP. In this study, we analyses the expression profiles of ncRNA by RNA sequencing, and predicted related functions through Gene Ontology (GO) and Kyoto Encyclopedia of Genes and Genomes (KEGG), then established the ceRNA network to discover the relationships between ncRNAs and mRNA. Our finding might discover new pathogenesis of SAP and provide new treatment ideas.

## Materials and Methods

### Animal Model and Sample Collection

Six male C57BL/6 mice aged 7–8weeks were purchased from Chengdu Dashuo Experimental Animal Technology Co., Ltd. living in independent ventilation cage (IVC) system and fed with standard food and water for 3days for environment adaptation. Animals fasted but were free to drink water the day before modeling. The next day, the mice were randomly divided into control group and SAP group (three mice per group). Before the operation, 5% isoflurane was given for inhalation anesthesia, and then 4% sodium taurocholate salt was slowly retrogradely injected into the biliopancreatic duct with a micro infusion pump to induce SAP. All animals were sacrificed after 24h. Collecting their pancreatic tissue and storing them at −80°C or fixing them in 4% paraformaldehyde for later use. The entire research plan has been approved by the Institutional Animal Care and Use Committee at the General Hospital of Western Theater Command.

### Analysis of Pancreatic Histology

The fixed pancreatic tissue was dehydrated with ethanol and embedded in paraffin. The 4um slice of pancreatic tissue was stained with hematoxylin-eosin. After dehydration and fixation, the slice was examined under the X200 optical microscope and the pictures were taken. Using the scoring system described previously (Schmidt et al., 1992), the score for each pancreatic tissue was averaged from every five microscopic vision.

### Amylase and Lipase Measurement

Blood was collected through the tail vein and serum levels of amylase and lipase were measured using a kit provided by the reagent manufacturer (Nanjing Jiancheng Bioengineering Research Institute, China).

### RNA Extraction and Quality Control

According to the manufacturer’s instructions, total RNA was extracted from frozen samples using Trizol reagent (Ambion), and the RNA concentration of each sample was measured using a NanoDrop ND-1000 instrument (Thermo Fisher Scientific, Waltham, MA, United States), with OD260/OD280 values as an indicator of RNA purity. RNA integrity was measured using denatured agarose gel electrophoresis.

### RNA Library Preparation and Sequencing

Ribo-Zero rRNA Removal Kits (Illumina, United States) was used to remove rRNAs from total RNA. RNA libraries were constructed by using rRNA-depleted RNAs with TruSeq Stranded Total RNA Library Prep Kit (Illumina, San Diego, CA, United States) according to the manufacturer’s instructions. Use Agilent DNA 1000 chip kit (Agilent, United States) to perform quality control and quantification of the library through BioAnalyzer 2100 system (Agilent Technologies, Inc., Santa Clara, CA, United States). 10pM libraries were denatured as single-stranded DNA molecules, captured on Illumina flow cells, amplified *in situ* as clusters and finally sequenced for 150cycles on Illumina HiSeq Sequencer according to the manufacturer’s instructions. Paired-end reads were harvested from Illumina HiSeq 4000 sequencer, and were quality controlled by Q30. After 3' adaptor-trimming and removing low quality reads by cutadapt software (version 1.9.3), the high-quality trimmed reads were used to analyze circRNAs, lncRNAs and mRNAs.

### RNA-Seq Analysis and Quality Assessment

After Illumina HiSeq 4000 sequencer sequencing, double-end reads were harvested. Use Q30 for quality control, use cutadapt (version 1.9.3; Martin, 2011) software to remove connectors and low-quality reads and then obtain high-quality reads.

#### Sequencing Analysis of lncRNA, circRNA and mRNA

The high-quality reads were aligned to the mouse reference genome (UCSC MM10) with Hisat2 software. Then, guided by the Ensembl gtf gene annotation file, Cuffdiff software (version 2.2.1; part of Cufflinks) was used to get the FPKM as the expression profiles of lncRNA and mRNA, and fold change and *p*-value were calculated based on FPKM, differentially expressed lncRNAs and mRNAs were identified (Trapnell et al., 2010). The high-quality reads were aligned to the reference genome/transcriptome with STAR software and circRNAs were detected and identified with DCC software (version 0.9.0). At the same time, circRNAs were annotated with CIRI softwore (version 1.1.1). Raw junction reads for all samples were normalized by total mapped reads number and log2 transformed. Calculating the fold change and *p*-value between the two groups of samples to screen for differential expression of circRNA.

#### Identification and Cluster Analysis of Differentially Expressed lncRNA, circRNA and mRNA

Use Cuffdiff software (version 2.2.1; part of Cufflinks software) to calculate and detect the differential expression of lncRNA (DEL) and mRNA (DEM) between the two groups. Using standardized reads, the differential expression of circRNA (DEC) between the two groups was calculated. The fold change ≥2.0, that is log (FC) ≥1.0, *p*-value < 0.05, and FPKM value in one sample at least >0.5 as the threshold for differential screening to identify differential genes. Using R’s heatmap2 function, cluster analysis of DEL and DEM was performed with FPKM value, and cluster analysis of DEC was performed with standardized reads. Using Venn diagrams, Volcano plots and Heat maps to show differential expression. The up- and down-regulated differential RNAs were represented by different color heat maps.

### Reverse Transcription Quantitative PCR

The results of RNA-seq need to be verified by RT-qPCR. Trizol is used to extract the total RNA in the pancreatic tissues of the control group and SAP group. The RNA is reverse transcribed into cDNA using SuperScriptTM III Reverse Transcriptase (Invitrogen) according to the manufacturer’s instructions. We used the QuantStudio 5 Real-Time PCR System (Thermo Fisher) for amplification with a total reaction volume of 10μl, including 0.5μl pre-PCR primer (10uM), 0.5μl post-PCR primer (10uM), 5μl 2 × Master Mix, 2μl cDNA and 2μl of enzyme-free water. The primers are listed in Table 1. The glyceraldehyde-3-phosphate dehydrogenase (GAPDH) was used as endogenous control. The PCR conditions were as follows: at 95°C for 10min, followed by 40cycles of PCR at 95°C for 10s and 60°C for 60s. The target RNA of the sample and the reference gene were measured through Real time PCR reaction. The data is analyzed by 2^-△△ CT^ method.

**Table 1:** Primer sequences for quantitative real-time polymerase chain reaction analysis of differentially expressed lncRNA and circRNA levels.

Name	Primer type	Primer sequence
AK004187	Forward	TGCAGTGTGAGTCTCCAGAG
	Reverse	GCTGAACGGAGAACAAGGAC
AK035396	Forward	GTGGAGGTCAGAAAGGGTCA
	Reverse	ACATCGACTCCCTTCCTCCT
AK162674	Forward	AGCATGCACACACTCTCTCT
	Reverse	ACAGATGGAGGACACAGGTG
NR_040328	Forward	GAGCCACCTGAATTACGCTG
	Reverse	AGTTAACAGGGCCCCAAACT
TCONS_00010866	Forward	GATGGTGAACTATGCCTGGG
	Reverse	GGCCCCAAGACCTCTAATCA
uc029sug.1	Forward	AACTCTGGTGGAGGTCCGTA
	Reverse	TCGGAGGGAACCAGCTACTA
CircAmy2a5	Forward	TGTTTTGGAGATTTGCTGTGAGA
	Reverse	GTCCTCACTTACCTAACAAAGAAAA
CircZFP644	Forward	CCGTTGATCTATCAGCCACA
	Reverse	GCACCAGTAATGTCGGTGTTT
CircDTNB	Forward	GCTCTGCCAGAACTGCTTTT
	Reverse	CCATTGGAATCTGACATCTGG
CircCELA3b	Forward	CCCCAGCAATAACATCGC
	Reverse	CAGACTGGATATCGCCGC
CircARHGEF38	Forward	CCTCCTCCGGGACTTGAT
	Reverse	CCCATGTCCAGCAGGTTC
CircSRPK2	Forward	TCAAGGCCTCCCAGTACG
	Reverse	GTGGTGGTGGTGGAGGAG
GAPDH	Forward	AAGGTCATCCCAGAGCTGAA
	Reverse	CTGCTTCACCACCTTCTTGA

### GO and KEGG Analysis

GO and KEGG were performed to predict the potential function of different lncRNA and circRNA. GO project^1^ has developed a set of structures controlled vocabulary for community annotation of genes, gene products and sequences, including three parts: Molecular Function (MF), Biological Process (BP) and Cell Component (CC). KEGG pathway is the process of mapping molecular data sets in genomics, transcriptomics, proteomics and metabolomics to the KEGG pathway map to explain the biological functions of these molecules. As we all know, lncRNA has a wide range of effects, and its mechanism of action is very complex, with multiple modes of action, including cis, trans, etc (Kopp and Mendell, 2018). There was evidence indicating that many lncRNAs act as cis regulators. Reportedly, the expression of lncRNAs was significantly related to the expression of protein-coding genes adjacent to their coordinates. The lncRNAs located upstream and downstream of the coding protein may overlap with promoters or other homeopathic elements co-expressing genes, so as to perform transcriptional activation and expression regulation of adjacent mRNA at the transcription or post-transcription level (Ulitsky and Bartel, 2013). Therefore, by searching for coding genes that were located 100kb upstream and downstream of the DEL and ensure that there was at least 1bp overlap with the DEL, as the target of the DEL. In organisms, under certain conditions, the transcript corresponding to the gene will form circular RNA and lose its coding function, thus affecting the biological phenotype. Therefore, we chose to analyze the host gene of circRNA. Consequently, GO and KEGG functional analysis was performed using the adjacent target genes of DEL, the host genes of circRNA and DEMs to annotate and speculate the functions of these differentially expressed genes. *p*-value < 0.05 is considered statistically significant and significantly enriched. Analyzing the data by double-sided FISHER precision test and calculating FDR to correct the *p*-value.

### Construction of lncRNA/circRNA -miRNA-mRNA Network

LncRNA and mRNA shares the sequence similarity, expression correlation, and positional neighbor relationship and both play a role through competing with miRNA molecules (Tay et al., 2014). And with the deepening of transcriptome research, it has been found that miRNA response element (MRE) exists on circRNA, which means that the same miRNA can bind to multiple types of RNA, and different RNA molecules that bind with same miRNA form a competitive relationship. We used Targetscan (Release 7.2) and Miranda (version 0.10.80) software to predict the relationship between miRNAs and DEMs, DELs and DECs, respectively, though base pairing, and integrated the predicted results to build the potential lncRNA/circRNA-miRNA-mRNA ceRNA network and use Cytoscape software (version 3.7.2) to visualize the results to explore the role of lncRNA/circRNA in the pathogenesis of SAP.

### Construction of PPI Network and Selection of Hub Genes

The hub gene is a gene that plays a vital role in the biological process. In related pathways, the regulation of other genes is often affected by the hub gene. The STRING database is an online database for searching known and predicted protein-protein interactions. These interactions include direct (physical) association and indirect (functional) association. Currently, the STRING database covers 24'584'628 proteins from 5'090 organisms. After introducing the up-regulated DEMs into the STRING database, we set the minimum interaction score as 0.900 (highest confidence) and hide disconnected nodes in the network to obtain a complex PPI network of DEMs. Then we use Cytoscape software (version 3.7.2) to visualize the PPI network, and use cytohubba (a plug-in of Cytoscape) to identify the most relevant nodes by setting the degree. If the setting degree is higher than 20, then the top 24 are considered as hub genes.

### Statistical Analysis

Statistical analyses were performed by the GraphPad Prism 7 (GraphPad, CA, United States) and SPSS 22.0 software packages (SPSS, IL, United States). Statistically significant differences between groups were estimated by Student’s *t*-test. Fisher’s exact test was applied in the GO and KEGG pathway analyses. The results were evaluated using Spearman’s correlation coefficient test. All values are expressed as the mean ± standard error of the mean; *p* < 0.05 was considered statistically significant.

## Result

### Evaluating the SAP Model

**Figure 1:**
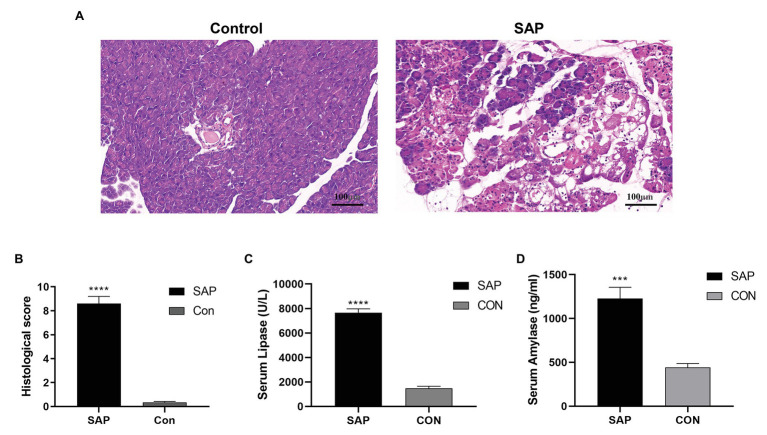
Evaluation of the SAP Model. **(A)** Compared with the control group (con), the histopathological sections of the pancreas in the SAP group showed focal expansion of the interlobular space of the pancreas, edema of the pancreatic lobules, extensive acinar cell necrosis and granulocyte infiltration (100x). **(B)** The histological score of pancreatic tissue. **(C,D)** The lipase and amylase levels were also significantly higher than the control group (*p* < 0.05). ^***^p < 0.001, vs. control group, *n* = 3 per group.

Compared with the control group, the HE staining of the pancreatic tissue in the SAP group showed typical histopathological changes, with focal expansion of the pancreatic interlobular septum, pancreatic lobular edema, extensive acinar cell necrosis and granulocyte infiltration (Figures 1A,B). At the same time, the serum lipase and amylase levels in the SAP group were also significantly higher than those in the control group (*p* < 0.05; Figures 1C,D), and the SAP model was successfully constructed.

### Overview of lncRNA, circRNA and mRNA Expression Profiles

**Figure 2:**
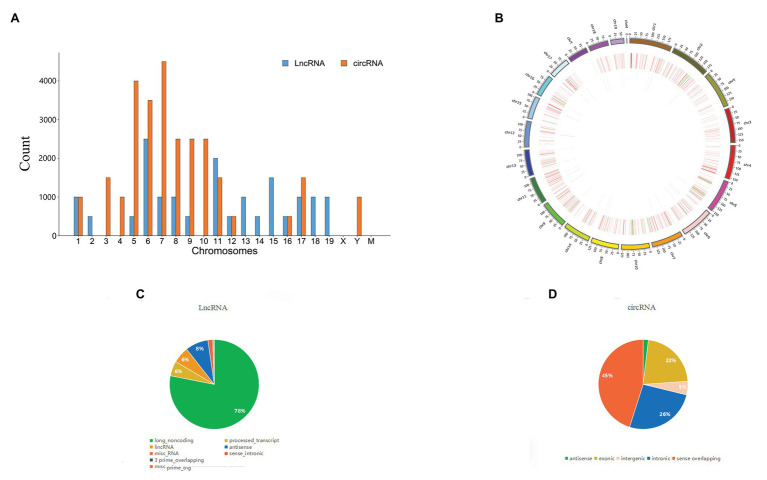
Comparison of the characteristics of lncRNA, circRNA, and mRNA expression profiles in pancreatic tissues between SAP group and control group. **(A)** The distribution of lncRNAs and circRNAs on chromosomes, **(B)** LncRNAs and mRNAs on chromosomes. **(C,D)** Biotypes of lncRNAs and circRNAs.

After removing redundant and low-quality reads, a total of 40.61 million and 39.94 million clean reads were obtained from the pancreatic head tissues of the control group and SAP group, respectively. A total of 56,348 lncRNAs, 6,350 circRNAs (Figure 2A) and 22,565 mRNAs (Figure 2B) were identified in all chromosomes. Among lncRNAs, most (78.1%) are long_noncoding RNA, 8.3% are antisense lncRNA, a few areintergenic lncRNA, processed_transcript lncRNA, and a very few are sense_intronic lncRNA (Figure 2C). In circRNA, the vast majority (45.1%) are sense overlapping, 26.1% are intronic, 22% are exonic, a few are intergenic, and a very small portion are antisense (Figure 2D).

### Differential Expression of lncRNAs, circRNAs and mRNAs

**Figure 3:**
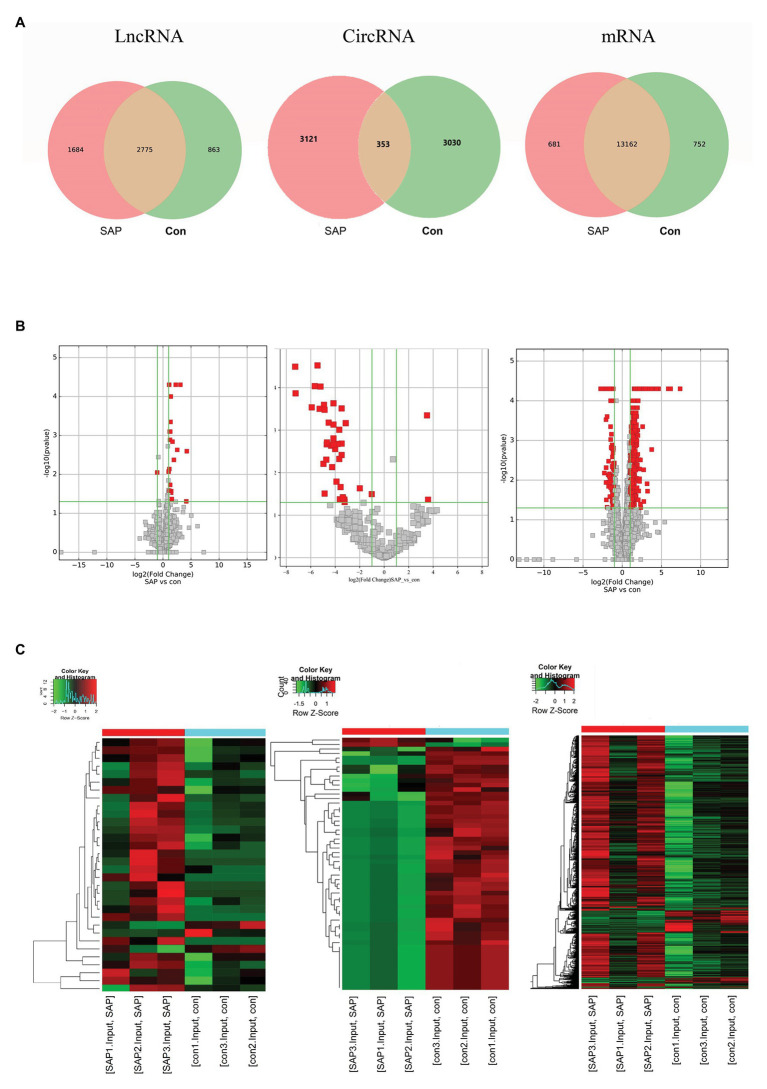
Differential expression of lncRNA, circRNA and mRNA. **(A)** The specific lncRNAs, circRNAs, and mRNAs shared between SAP group and control (con) group. The red points showed the up-regulated lncRNAs, circRNAs, mRNAs. The green points showed the down-regulated lncRNAs, circRNAs, and mRNAs. **(B)** The volcano diagrams showed the DE lncRNAs, circRNAs and mRNAs in the pancreatic head tissue of the three pairs of SAP group and control group, respectively. **(C)** Heatmap showing DE lncRNAs, circRNAs and mRNAs from pancreatic tissues of SAP mice compared to pancreatic tissues of control mice, respectively. Row and column represent DE lncRNA/DE circRNA/DE mRNA transcripts and tissue samples, red color represents up-regulated DERNAs, green color represents down-regulated DERNAs, and heavier color represents higher fold change.

In order to fully understand the pathogenesis of SAP, we simultaneously analyzed the profiles of DEL, DEC and DEM through microarray. Significant difference is defined as fold change ≥ 2 and *p* < 0.05. In this study, the SAP group identified 49 lncRNAs, 56 circRNAs and 1,194 mRNAs with significant differential expression. The control group and SAP group shared 5% of lncRNAs, 5% of circRNAs and 90% of mRNAs (Figure 3A). Compared with the control group, the SAP group identified 46 up-regulated and 3 down-regulated lncRNAs, 2 up-regulated and 54 down-regulated circRNAs, and 1,053 up-regulated and 141 down-regulated mRNAs. The Volcano map (Figure 3B) and hierarchical cluster analysis (Figure 3C) of lncRNA, circRNA, and mRNA showed significant differences of pancreatic tissues between control group and SAP group.

### Verify Differentially Dysregulated lncRNAs and circRNAs

**Figure 4:**
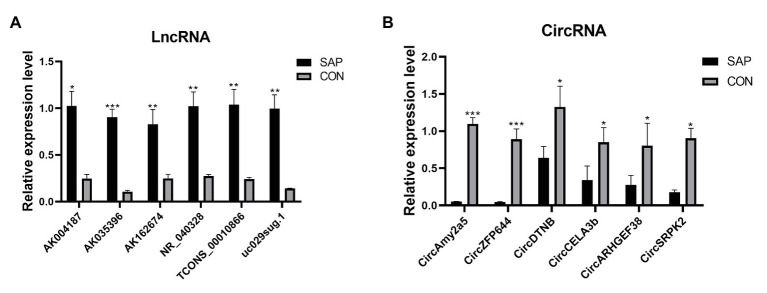
qRT-PCR validation of DELs and DECs in SAP rats compared with matched tissues of control rats. **(A)** The expression level of lncRNA AK004187, AK035396, AK162674, NR_040328, uc029sug.1, TCONS_00010866. **(B)** The expression level of circRNA CircAmy2a5, CircZFP644, CircDTNB, CircCELA3b, CircARHGEF38, and CircSRPK2. ^*^represents *p* < 0.05, ^**^represents *p* < 0.01, and ^***^represents *p* < 0.001.

Based on the selected DELs, 46 lncRNAs were up-regulated and 54 circRNAs were down-regulated. We focus on the up-regulated lncRNAs that have more significant changes. For further research, we selected 6 up-regulated lncRNAs and 6 down-regulated circRNAs for qRT-PCR verification according to the standard of lncRNA/circRNA with approximately the same length, *p* value < 0.05, Fold change ≥2. Compared with control group, the lncRNA selected in SAP group, including AK004187, AK035396, AK162674, NR_040328, uc029sug.1, TCONS_00010866, were significantly overexpressed and consistent with the RNA Sequencing results (Figure 4A). circRNA selected in SAP group, including CircAmy2a5, CircZFP644, CircDTNB, CircCELA3b, CircARHGEF38, and CircSRPK2, were significantly down regulated by comparing with control group and consistent with the RNA Sequencing results (Figure 4B). The primers are listed in Table 1. Therefore, these all proved the accuracy of the microarray results.

### GO and KEGG Analysis of Differentially Expressed lncRNAs and circRNAs

**Figure 5:**
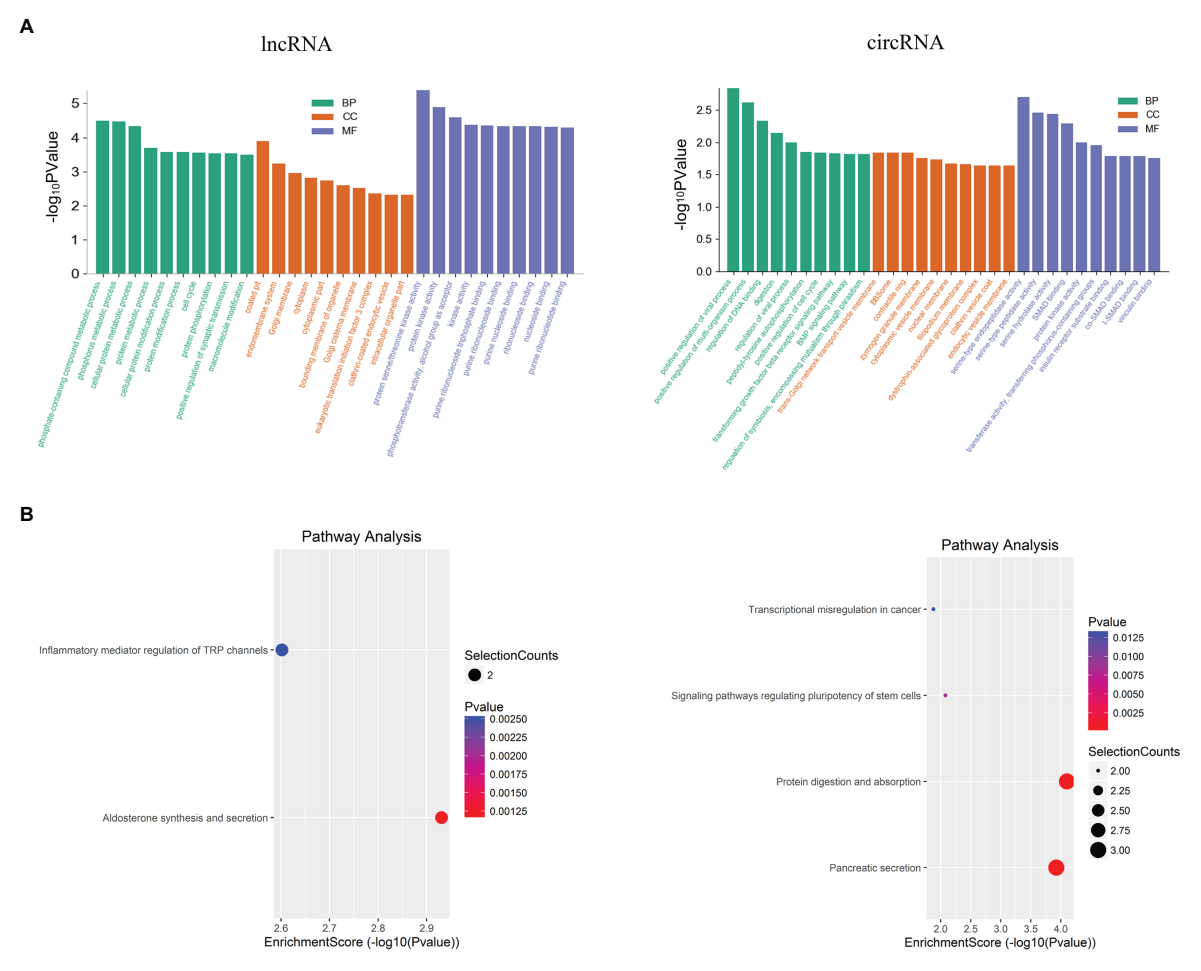
Functional analysis for the differentially expressed mRNAs, lncRNAs and circRNAs. **(A)** GO enrichment analysis for up-regulated mRNAs, up-regulated lncRNAs and the down-regulated circRNAs. Red bars are biological process, green bars are cell component, and blue bars are molecular function. The ordinate is -Log10 *p* value (-LgP). The larger the -LgP value, the smaller the P value, indicating that the enrichment of differentially expressed mRNA/lncRNA/circRNA in a given pathway is more significant. **(B)** KEGG pathway enrichment analysis for up-regulated mRNAs, up-regulated lncRNAs, and for down-regulated circRNAs. Size represents the number of enriched genes, and color indicates the degree of enrichment. Higher enrichment scores correlate with lower *p*-value, indicating that the enrichment of differentially expressed genes in the given pathway is significant.

The functions of DELs and DECs were analyzed by GO and KEGG. The function predictions of GO in DEL focus on CC are related to the formation of Golgi membrane and clathrin vesicle endocytosis. The function predictions focus on MF are related to regulate eukaryotic translation initiation factor 3 (ETIF3) activity, calcium-dependent protein kinase activity, PKC activity and MAPKKK activity (Figure 5A). The GO analysis of DEC showed that the functional predictions of target genes were mainly enriched in CC and MF. Among them, DEC is related to Golgi network transport vesicle membrane and zymogen granules, and participates in serine endopeptidase, hydrolase activity and amylase activity (Figure 5A). KEGG analysis revealed the potential mechanism of DEL and DEC in SAP (Figure 5B). DEL participates in the regulation of inflammatory mediators of TRP channels. The parent gene of DEC might play parts in protein digestion and absorption, pancreatic secretion, regulation of stem cell versatility, and transcriptional disorders.

### Construction of lncRNA/circRNA-miRNA-mRNA Network

**Figure 6:**
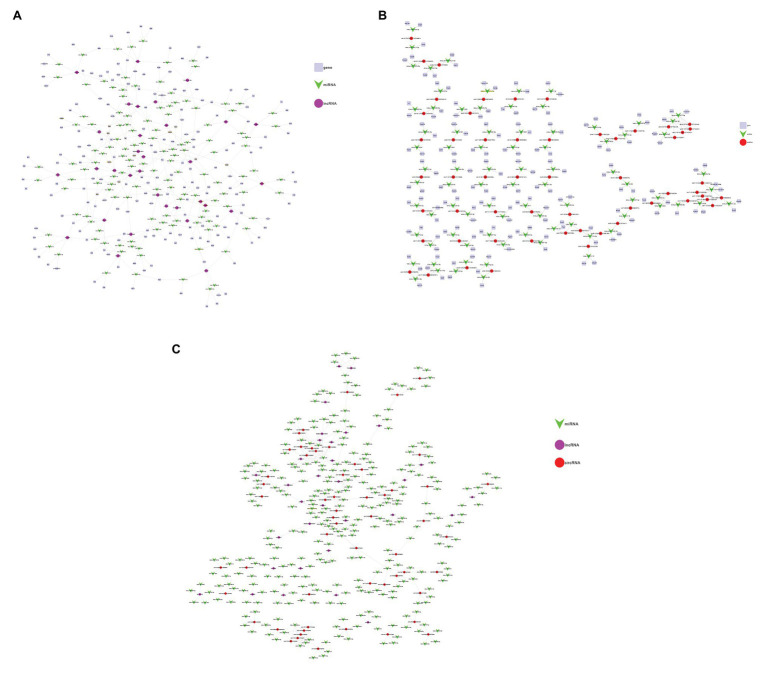
Construction of the lncRNA/circRNA-miRNA-mRNA Network. **(A)** Network analysis of lncRNA-miRNA-mRNA. The square nodes represent mRNA. The triangle node represents miRNA. The round node represents lncRNA. **(B)** Network analysis of circRNA-miRNA-mRNA. The square nodes represent mRNA. The triangle node represents miRNA. The round node represents circRNA. **(C)** Network analysis of lncRNA-circRNA-miRNA. The purple nodes represent lncRNA. The green node represents miRNA. The red node represents circRNA.

LncRNA participates in the regulation of biological processes in different ways. It can directly combine with mRNA to change the spatial conformation of chromatin. As a result, the expression level of mRNA is change (Marchese et al., 2017). It can also be interfered by regulatory molecules. Based on the relationships between lncRNA and mRNA and the role of miRNA as a middle regulatory molecule, we constructed a lncRNA-miRNA-mRNA ceRNA network to predict the functions of DEL and DEM. As shown in the figure below (Figure 6A), the network involves 32 lncRNAs, 186 mRNAs and 133 miRNAs. At the same time, a circRNA-miRNA-mRNA ceRNA network was constructed (Figure 6B), involving 56 circRNAs, 155 mRNAs and 82 miRNAs. Each differentially expressed gene can be associated with one or more miRNAs. For example, lncRNA AK004187 has established connections with 5 miRNAs, including miR-7222-5p, miR-6540-5p, miR-6972-3p, miR-6991-5p, miR-3078-5p. miR-1187 has been connected with 6 circRNAs, including CircDmbt1 and CircZfp644. And CircARHGEF only established a connection with miR-710. The two networks have multiple common nodes (Figure 6C), such as lncRNA AK162674, lncRNA AK028540, lncRNA uc008hmc.1, CircDmbt1, and CircZfp644, which all interact with miR-1187 to regulate Notch signaling; lncRNA uc029rzf.1, lncRNA uc007jfe.1, CircZfp644 and CircTCRB interacts with miR-6,952 and regulates EPS8; lncRNA AK162674, lncRNA ENSMUST00000128078, CircErdr1, CircDmbt1 and CircMuc6 interact with miR-466i-5p to regulate the expression of Rorc; lncRNA uc007hxv.1, lncRNA ENSMUST00000182761 and CircSrpk2 can link to miR-7081-5p and then affect the expression of Tmem38b to regulate intracellular calcium release. The ceRNA network shows that these lncRNAs and circRNAs can not only independently participate in the regulation of biological processes, but also act as miRNA sponge together to affect downstream gene expression and participate in the occurrence and development of SAP.

### PPI Network and Hub Genes Analysis

**Figure 7:**
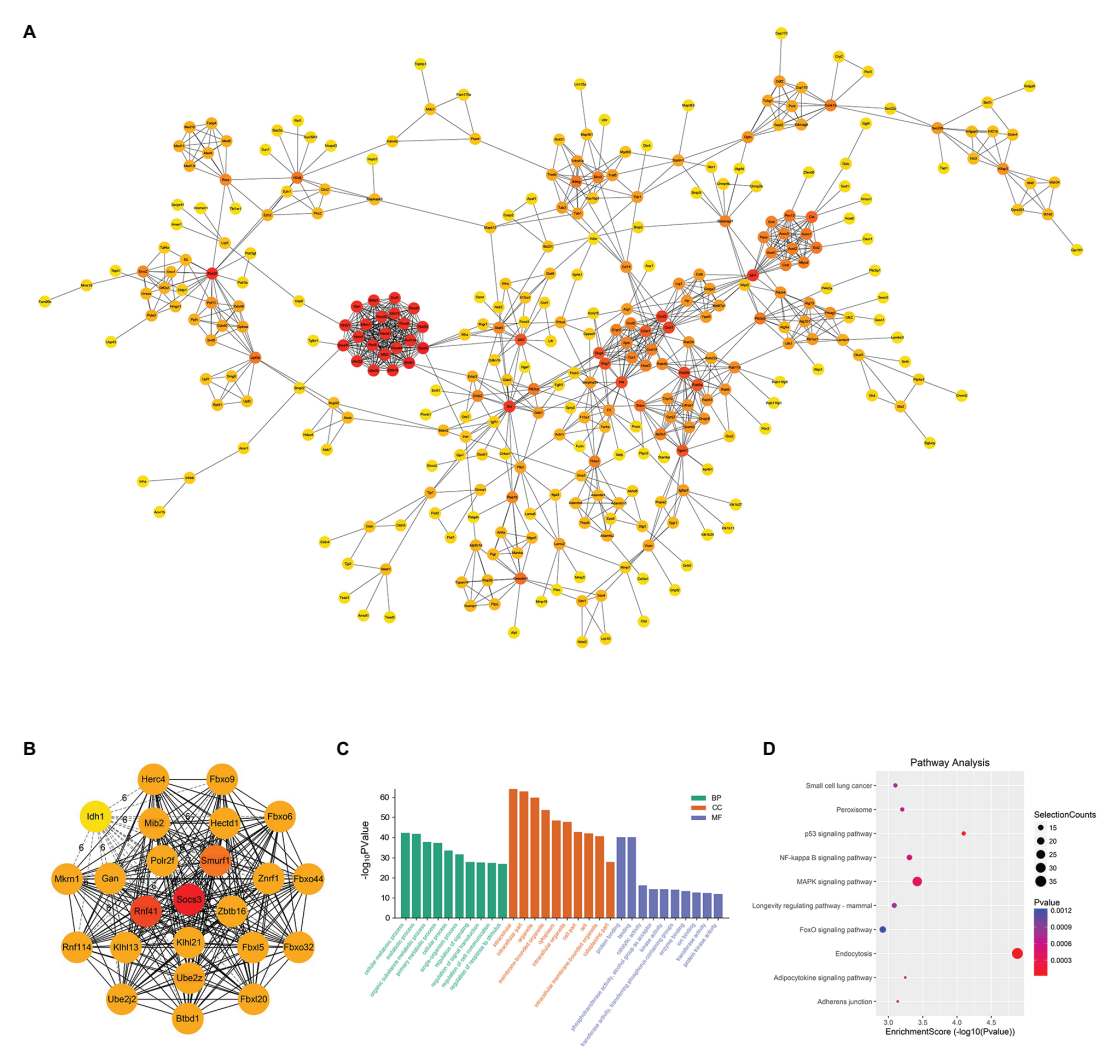
PPI Network and Hub Gene Analysis. **(A)** Import the up-regulated DEMs into the STRING database to build a PPI network with 433 nodes and 1,137 edges. The darker the red of the circle means that the gene has a higher degree and has more connections with other genes; conversely, the closer the color of the circle is to yellow, the less the degree of the gene is, and the less the connection with other genes. **(B)** Recalculate the degree of 24 DEMs with degree >20 and get the top five genes as hub genes. These hub genes are Socs3 (degree = 26), Rnf41 (degree = 24), Smurf1 (degree = 23), Mib2 (degree = 21) and Ube2z (degree = 21). **(C)** GO analysis of DEMs. **(D)** KEGG analysis of DEMs.

When constructing the PPI network, we set the minimum interaction score as 0.900 and calculated the degree to obtain a PPI network with 433 nodes and 1,137 edges (Figure 7A). We copied the module with degree >20 and used the Cytohubba plug-in to calculate the degree again to get the top 24 genes (Figure 7B). The genes with the highest connectivity were hub genes, including Socs3 (degree = 26), Rnf41 (degree = 24), Smurf1 (degree = 23), Mib2 (degree = 21), Ube2z (degree = 21). In order to clarify the role of differential genes in SAP, we performed GO and KEGG analysis on DEMs It is predicted that the function is mainly enriched in the BP signal transduction, cell communication and the regulation process of stimulus response. In CC, functions are highly enriched in autophagy-related processes, which are related to the assembly of autophagic vesicles and the structure of autophagy precursors and participate in the activation of transforming factor β (TGF-β) receptors in MF (Figure 7C). KEGG analysis showed DEM was involved in endocytosis, P53 signaling pathway, MAPK signaling pathway, and NF-κB signaling pathway (Figure 7D). These results showed that most of the hub genes play a role in different pathogenesis of SAP.

## Discussion

In order to explore the underlying function of lncRNA and circRNA in SAP, in this study, we firstly performed the expression profiles of lncRNA, circRNA and mRNA by RNA-seq analysis. In total, we identified 56,348 lncRNAs, 6,350 circRNAs and 22,565 mRNAs. Among them, 46 lncRNAs were up-regulated and 3 lncRNAs were down-regulated, 2 circRNAs were up-regulated and 54 circRNAs were down-regulated, and 1,053 mRNAs were up-regulated and 141 mRNAs were down-regulated. Interestingly, we found that the expression of lncRNAs and mRNAs are consistent in trend, and the main trend is that more transcripts are up-regulated. Feng et al. also found that the expression trends of lncRNAs and mRNAs are consistent when they predicted endometrial receptivity by analyzing co-expression of lncRNAs-mRNAs (Feng et al., 2018). The reason may be that lncRNAs and mRNAs are similar, related and can be directly regulated (Zhang et al., 2019a). Then, we selected 6 lncRNAs from the most up-regulated lncRNAs and 6 circRNAs from the most down-regulated circRNAs for qRT-PCR low-throughput verification, and the results were consistent with the sequencing results. Importantly, in lncRNA/circRNA-miRNA-mRNA ceRNA networks, we found that these DEMs, DELs and DECs are involved in the key process of SAP, such as apoptosis pathway and calcium channel regulation. It indicated that ncRNAs may play an important role in SAP. In the PPI network, the GO and KEGG analysis of the top five hub genes showed these hub genes were mainly involved in inflammation regulation pathways, cell polarization processes, apoptosis and Notch pathways, and these mechanisms were all closely related to the occurrence of SAP. Our findings may provide new insights into understanding the function of mRNA/lncRNA/circRNA in the pathophysiology of SAP.

Currently, a number of accumulating evidence suggests that ncRNAs, especially lncRNAs and circRNAs, play an important role in process of many diseases *via* different ways, such as coronary artery disease (Li et al., 2018), inflammatory bowel disease (Liu et al., 2018), ankylosing spondylitis (Li et al., 2017), kidney injury (Liu et al., 2019), pancreatic cancer (Deng et al., 2018a). Firstly, lncRNA participates in important signal transduction pathways by binding to proteins, interfering with the action of protein-modifying enzymes, and activating dependent kinase activity. For example, in PTEN-deficient prostate cancer, lncRNA PCAT1 directly binds to FKBP51 and replaces PHLPP in the PHLPP/FKBP51/IKKα complex, thereby activating AKT and NF-κB signals and accelerating the progression of the disease (Shang et al., 2019). Besides, lncRNA NKILA can bind to NF-κB/IκB and inhibit IKK-mediated phosphorylation of IκB by directly masking/covering phosphorylation sites (Liu et al., 2015). And Sang et al. found that lncRNA CamK-A activated calmodulin-dependent kinase PNCK under hypoxic conditions, which in turn caused IKBa phosphorylates and triggered the calcium-dependent nuclear factor NF-κB, finally resulting in the inflammatory response (Sang et al., 2018). Secondly, lncRNA can act as a sponge of miRNA to regulate target mRNA. Li and his colleagues determined the role of lncRNA as an autophagy promoting factor (APF) in myocardial infarction. They found that lncRNA APF reduced the inhibitory effect of miR-188-3p on the target autophagy-related protein 7(ATG7) by sponging miR-188-3, and then promoted autophagy and cell death (Wang et al., 2015). In this study, we found that lncRNA as a sponge of miRNA mainly participates in the progress of SAP through regulation of acinar cell apoptosis and calcium release. On the one hand, in the lncRNA ceRNA network, lncRNA AK004187 acts as a sponge of miR-6991-5p to regulate the target gene Bmf, and lncRNA uc029sug.1 acts as the sponge of miR-1968-3p to regulate Gab1. It has confirmed that Bmf can function as apoptosis activator (Hayakawa et al., 2016) and Gab1 can plays a central role in cell growth, transformation and apoptosis (Zhang et al., 2019b). Reportedly, apoptosis has protective effects on SAP (Luo et al., 2020). Therefore, we speculate that lncRNA AK004187 and lncRNA uc029sug.1 could influence the severity of the disease by regulating the apoptosis of acinar cells in SAP. On the other hand, pathologically elevation of Ca^2+^ concentration in acinar cells is a central event in AP, which mediates pro-cell death and pro-inflammatory pathways. Cabral et al. found that Tmem38b gene could maintain intracellular calcium release (Cabral et al., 2016). In lncRNA ceRNA network, lncRNA uc007hxv.1 and lncRNA ENSMUST00000182761 both can act as sponges of miR-7081-5p, regulating the expression of Tmem38b and the release of intracellular calcium.

In addition, we found that lncRNA is also involved in other pathways in our results. LncRNA AK162674 and lncRNA ENSMUST00000128078 act as sponges of miR-466i-5p to regulate the expression of Rorc. The Rorc gene is a part of the molecular cascade that regulates insulin secretion in pancreatic β-cells. Silencing Rorc by siRNA in INS-1 (832/13) cells leads to a significant down-regulation of insulin mRNA expression and insulin secretion (Taneera et al., 2019). We speculated whether the effect of lncRNA on Rorc gene expression has a potential therapeutic effect on pancreatic endocrine disorders. Of note, we found that lncRNA uc029rzf.1, lncRNA ENSMUST00000182761 and lncRNA TCONS_00027293 can regulate the expression of epidermal growth factor receptor (EGFR), epidermal growth factor receptor pathway substrate (EPS) and transforming growth factor beta (TGF-β) genes. EPS contains a PH domain and an SH3 domain. Although its exact role has not been determined, it works as part of the EGFR pathway involved in cell proliferation (Ding et al., 2008). LncRNA uc029rzf.1 can directly act on EPS, while lncRNA ENSMUST00000182761 regulates EPS8 through sponging miR-7092-3/being a miR-7092-3 sponge. TGF-β can regulate cell proliferation, differentiation and growth and expression and activation of other growth factors (including interferon gamma and tumor necrosis factor alpha) and exerts an important impact on tumors and inflammatory diseases (Tözser et al., 2015). LncRNA TCONS_00027293 acts as a sponge of miR-574-3p to regulate the expression of TGF-β to affect the occurrence of tumors. However, the specific role of these lncRNAs in SAP needs to be further studied.

Importantly, lncRNA and circRNA can both regulate the expression of genes *via* sponging miRNA. We found that these two networks share two important nodes, Notch signaling and EPS signaling, in SAP by establishing the lncRNA/circRNA-miRNA network. The first is Notch. Notch signaling is an evolutionarily conserved intercellular signaling pathway, which can regulate the interaction between physically adjacent cells by combining Notch family receptors with their cognate ligands. The encoded pre-protein undergoes proteolysis in the anti-Golgi network to generate two heterodimers to form the polypeptide chains of mature cell surface receptors. This receptor plays a role in the development of many cell and tissue types (Zhang et al., 2019c). lncRNA AK162674 and CircDMBT1 can act as sponges of miR-1187 and regulate Notch signaling. The second is EPS. Both CircZFP644 and lncRNA uc007jfe.1 can be used as ceRNA to competitively bind with miR-6952-3p to regulate EPS. From these data, it can be inferred that lncRNA and circRNA may involve in the pathogenesis of SAP together.

Of course, circRNA can also be combined with miRNA alone to regulate downstream target genes. Our previous study has found that CircZFP644 may act as a sponge for miR-21-3p and regulate its target genes, ultimately leading to the occurrence of necrotizing pancreatitis (Yang et al., 2020). In circRNA-miRNA-mRNA ceRNA network, we found that CircZFP644, CircDTNB, and CircARHGEF participate in the development of SAP by regulating calcium signal transduction, and CircSRPK2 participates in the development of SAP by regulating apoptosis and autophagy. CircZFP644 can act as a sponge of miR-6929-3p to regulate the target gene Cdh22, which encodes cadherin (Kelly et al., 2018). As the sponge of miR-6938-3p, CircDTNB could regulate Calb1 to play the role in calcium-binding proteins including calmodulin and troponin (Cao et al., 2019). At the same time, CircARHGEF acts as a sponge of miR-710 to regulate Atp2c1, thereby affecting calcium ion transport (Deng et al., 2018b). However, the roles of CircZFP644, CircDTNB, and CircARHGEF involved in SAP pathological calcium signal transduction still needs further verification. Gulp1 is an adaptor protein necessary for cell phagocytosis and apoptosis (Maldonado et al., 2018). CircSRPK2 acts as a sponge of miR-653-3p to regulate Gulp1, which may have a potential effect on apoptosis in SAP.

Additionaly, we constructed a PPI network and screened out 5 hub genes in the DEMs that were up- regulated in the SAP group, which may play a key role in SAP. These hub genes were Socs3, Rnf41, Smurf1, Mib2, and Ube2z. Some studies had shown that pancreatitis-associated protein (PAP) was found in the pancreatic juice of AP rats, which can activated the expression of Socs3 through JAK/STAT3-dependent pathways to exert anti-inflammatory effects (Closa et al., 2007). At the same time, Socs3 deficiency promotes the polarization and inflammation of M1 macrophages (Qin et al., 2012). Moreover, Rnf41 can also function through the activated JAK-STAT pathway, acting as a key regulator of cytokine receptors to maintain the level of basic cytokine receptors (Wauman et al., 2011; De Ceuninck et al., 2013). Cao et al. reported that Smurf1 was associated with a number of important biological pathways, such as the Wnt pathway, cell division, cell growth, and autophagy (Cao and Zhang, 2013). And these biological pathways were related to the occurrence of SAP. Mib2 can enhance NF-κB signaling in inflammatory response by promoting ubiquitin-dependent CYLD degradation in the Notch pathway (Uematsu et al., 2019). According to reports, Ube2z was a member of ubiquitin-conjugating enzymes and was involved in a variety of biological processes. Reportedly, Ube2z may promoted hepatocellular carcinoma process by targeting ERK and stat3 signaling pathways (Shi et al., 2020). More importantly, the results of GO and KEGG analysis in this study show that the most important GO and KEGG terms related to the up-regulated DEMs are also related to inflammation regulation pathways, endocytosis, cell interaction and Notch pathways. These results suggest that these genes play an important role in the pathogenesis of SAP.

Furthermore, in GO and KEGG analysis of DELs and DECs, they are enriched in these pathways, including calcium regulation signaling pathway, NF-κB signaling pathway, autophagy and protein digestion and absorption process, suggesting that lncRNA and circRNA may involve in the pathogenesis of SAP. The dysregulation of the calcium-regulated signaling pathway is the central event that occurs in SAP and mediates the activation of the NF-κB signaling pathway (Berry et al., 2018). At the same time, in SAP, NF-κB is the core molecule that links the initial acinar damage with systemic inflammation and continues inflammation (Jakkampudi et al., 2016). The unobstructed autophagy process is the basis for cells to maintain their metabolic functions, and the abnormal autophagy can cause autophagic vesicles to accumulation and lysosome inactivation. Acinar cells are highly efficient in protein production, and are easily affected by the endoplasmic reticulum stress, finally leading to unfolded protein response and so on (Wu et al., 2016). What’s more, endoplasmic reticulum stress (Biczo et al., 2018) and unfolded protein response (Meyerovich et al., 2016) all promote the progress of SAP. These reports and our results also suggest that lncRNA and circRNA play important roles in the pathogenesis of SAP.

All in all, our research provides a preliminary overview of the differentially expressed lncRNA, circRNA and mRNA in SAP for the first time, which may provide novel insights into understanding the pathogenesis of SAP and help seek new therapeutic targets and provide ideas for clinical treatment. The lncRNA/circRNA-miRNA-mRNA ceRNA network and the PPI network only provide us with possible mechanisms to understand SAP, but further *in vivo* and *in vitro* experiments are urgently needed to study and confirm the role of lncRNA and circRNA in SAP.

## Data Availability Statement

The datasets presented in this study can be found in online repositories. The names of the repository/repositories and accession number(s) can be found at: https://www.ncbi.nlm.nih.gov/geo/query/acc.cgi?acc=GSE161945.

## Ethics Statement

The animal study was reviewed and approved by Institutional Animal Care and Use Committee at the General Hospital of Western Theater Command.

## Author Contributions

HS, LT, BW, and JW conceived and designed the study. QH constructed the SAP model. XY, YY, WJ, and YW conducted experiments and analyzed the data. BW and JW wrote the main manuscript and prepared all table and figures. LT and HS carefully reviewed and proofread the manuscript. All authors have reviewed the manuscript before submitting it and approved the final version of the manuscript.

### Conflict of Interest

The authors declare that the research was conducted in the absence of any commercial or financial relationships that could be construed as a potential conflict of interest.

## Footnotes

^1^http://www.geneontology.org
