# Supplementary figures and images for: Comprehensive Analysis of Differentially Expressed lncRNA, circRNA and mRNA and Their ceRNA Networks in Mice With Severe Acute Pancreatitis
Source: Front Genet. 2021 Jan 28;12:625846. doi: 10.3389/fgene.2021.625846 (PMC7876390; doi:10.3389/fgene.2021.625846)

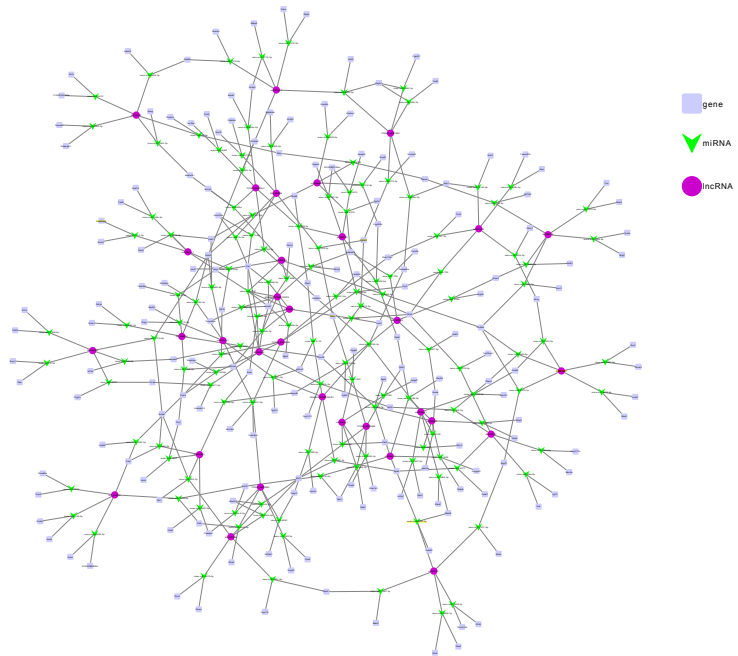

Supplement: Supplementary file 1 [file Data_Sheet_1.pdf]

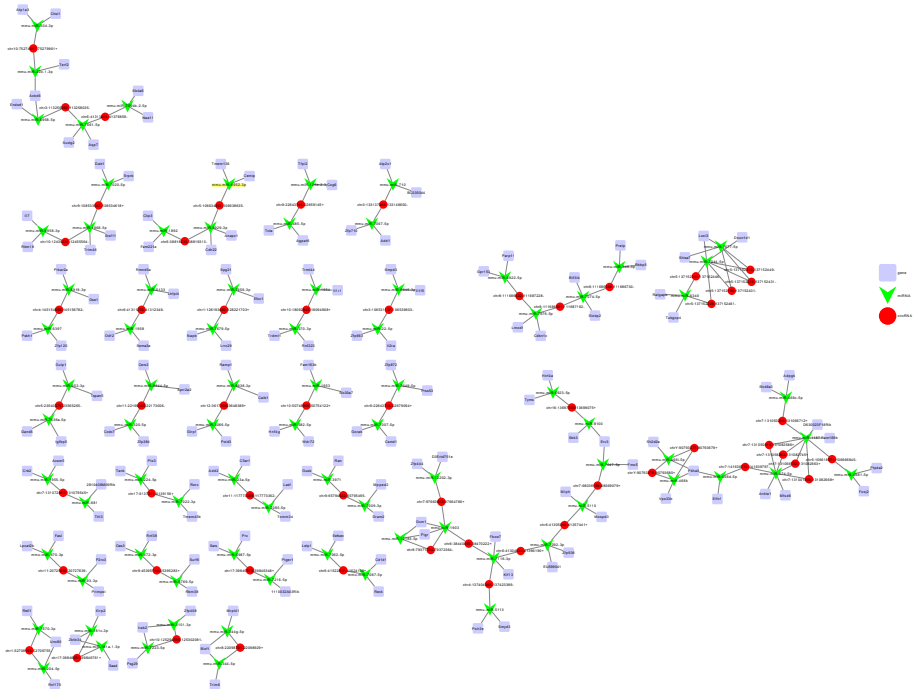

Supplement: Supplementary file 2 [file Data_Sheet_2.pdf]

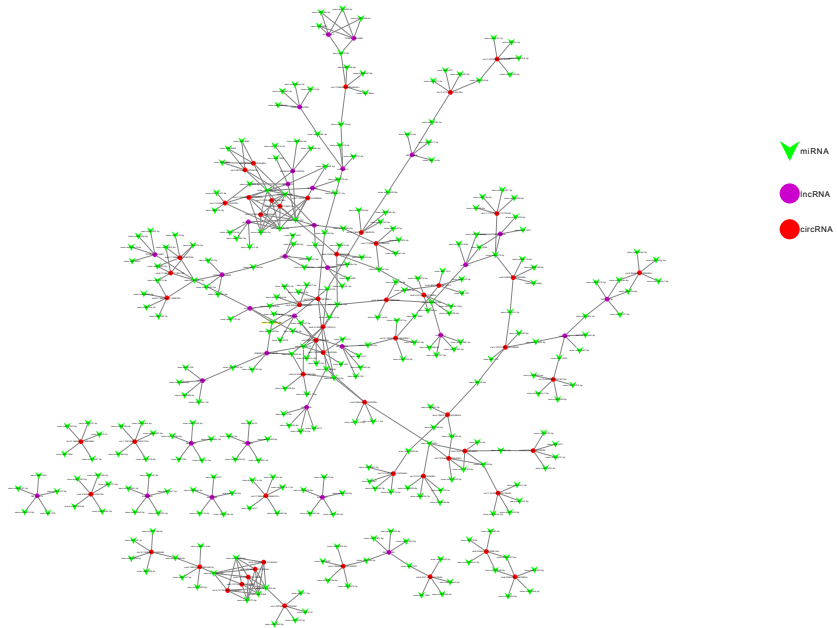

Supplement: Supplementary file 3 [file Data_Sheet_3.pdf]
